# Comparison of HPV genotyping and methylated *ZNF582* as triage for women with equivocal liquid-based cytology results

**DOI:** 10.1186/s13148-015-0084-2

**Published:** 2015-04-28

**Authors:** Yu-Ligh Liou, Yu Zhang, Yingzi Liu, Lanqin Cao, Chong-Zhen Qin, Tao-Lan Zhang, Chi-Feng Chang, Huei-Jen Wang, Shu-Yi Lin, Tang-Yuan Chu, Yi Zhang, Hong-Hao Zhou

**Affiliations:** Department of Clinical Pharmacology, Xiangya Hospital, Central South University, No. 110 Xiangya Road, Changsha, Hunan 410008 People’s Republic of China; Department of Obstetrics and Gynecology, Xiangya Hospital, Central South University, No. 87 Xiangya Road, Changsha, Hunan 410008 People’s Republic of China; Institute of Clinical Pharmacology, Central South University, Hunan Key Laboratory of Pharmacogenetics, No. 110 Xiangya Road, Changsha, Hunan 410078 People’s Republic of China; Department of Obstetrics and Gynecology, Buddhist Tzu Chi General Hospital, No.707, Sec. 3, Zhongyang Rd., Hualien, 97002 Taiwan; iStat Biomedical Co. Ltd., 18F, No. 96, Sec.1, Xintai 5th Road, Xizhi Dist., Taipei, 22102 Taiwan; Department of Education and Research, Taipei City Hospital, No.10, Sec. 4, Ren’ai Rd., Da’an Dist., Taipei, 10629 Taiwan; Institure of Medical Science, Tzu Chi University, No.707, Sec. 3, Zhongyang Rd., Hualien, 97002 Taiwan; Center for Cervical Cancer Prevention, Department of Research Buddhist Tzu Chi General Hospital, No.707, Sec. 3, Zhongyang Rd., Hualien, 97002 Taiwan

**Keywords:** Biomarker, DNA methylation, HPV-HR, HPV-16/18, Cervical cancer, *ZNF582*

## Abstract

**Introduction:**

The interpretation of equivocal Papanicolaou (Pap) smear results remains challenging, even with the addition of the high-risk human papillomavirus test (HPV-HR). Recently, methylated zinc finger protein 582 (*ZNF582*) (*ZNF582*^*m*^) was reported to be highly associated with cervical cancer. In this study, we compared the performance of *ZNF582*^*m*^ detection and HPV-HR genotyping in the triage of cervical atypical squamous cell of undetermined significance (ASC-US) and atypical squamous cell - cannot exclude a high-grade lesion (ASC-H).

**Case description:**

Two hundred and forty-two subjects with equivocal papanicolaou smear (Pap smear) results were recruited in this hospital-based and case-controlled study. The residual cervical cells in liquid-based cytological test (LBC) containers were used for genomic DNA extraction and then for *ZNF582*^*m*^ and HPV-HR detection. The level of *ZNF582*^*m*^ was quantified by real-time methylation-specific PCR after bisulfite conversion. The HPV-HR test was performed by using a nested multiplex PCR (NMPCR) assay that combines degenerate E6/E7 consensus primers and HPV type-specific primers.

**Discussion and evaluation:**

Significant associations were observed between *ZNF582*^*m*^ and the risk of cervical intraepithelial neoplasia grade 3 or higher (CIN3+; odds ratio = 15.52, 95% confidence interval (CI): 7.73 to 31.18). The sensitivity and specificity of *ZNF582*^*m*^ for women with CIN3+ were 82.43% and 76.79%, respectively. High sensitivity (99.33%) but low specificity (38.76%) was observed for HPV-HR. When combining both positive results of *ZNF582*^*m*^ and HPV-HR, the sensitivity and specificity were 82.43% and 81.55%, respectively. The sensitivity and specificity of *ZNF582*^*m*^ or HPV-16/18 were 89.19% and 70.24%, respectively. However, the sensitivity and specificity of *ZNF582*^*m*^ combined with HPV-16/18 (both *ZNF582*^*m*^ and HPV-16/18 positive results) were 59.46% and 94.64%, respectively.

**Conclusions:**

*ZNF582*^*m*^ provides a promising triage tool for women with ASC.

To effectively manage ASC patients, a new strategy co-testing for *ZNF582*^*m*^ and HPV-16/18 genotyping was proposed. This strategy could reduce the number of patients referred for colposcopic examination and thus provide a feasible follow-up solution in the regions where colposcopy is not readily available. This strategy could also prevent women from experiencing unnecessary anxiety caused by HPV-HR.

## Background

Cervical cancer is the fourth most common cancer that affects women worldwide. In China, approximately 58,000 new cases and 20,000 deaths were documented in 2005 [1-5]. Screening with the cytological test developed by George Papnicolaou (the Pap test) has led to a significant reduction in the incidence of cervical cancer over the past few decades. However, the optimal treatment regimen for women with atypical squamous cell (ASC) is not well established. In the case of an ASC smear result, a pathologist has to specify the type as either atypical squamous cell of undetermined significance (ASC-US) or cannot exclude a high-grade lesion (ASC-H). ASC-US and ASC-H need to be closely monitored to prevent the development of high-grade squamous intraepithelial lesions (HSIL). Hybrid capture 2 human papillomavirus (HC2 HPV) testing has been widely adopted for the triage of patients with ASC-US [6]. The recommendation for ASC-H is colposcopy [7]. The sensitivity of HPV testing is good; however, the high prevalence of transient HPV infections limits the specificity of this approach, resulting in high number of colposcopic referrals and unnecessary anxiety for women. Thus, there is a need for other markers, which have both high sensitivity and specificity [8-10].

The mechanisms of pathogenesis in cervical cancer are not entirely clear. Hypermethylation of CpG sites in promoter regions silences the transcription of tumor suppressor genes and is an early and crucial event in cancer development [11-13]. Inactivation of tumor suppressor genes and activation of oncogenes also play a significant role in carcinogenesis, caused by genetic and epigenetic alterations. Various genome-wide approaches have been used to discover new abnormally methylated genes for many cancers. For example, candidate methylated genes have been selected, including *COL25A1* and *KATNAL2*, which showed increased methylation during progression in high-grade cervical intraepithelial neoplasia (CIN) lesions, to screen for the early detection of cervical cancer [14,15]. The advanced concept of using DNA methylation as a biomarker for cervical cancer screening is appealing [16-19], and several hypermethylated genes have already been identified [20]. Among them, zinc finger protein 582 (*ZNF582*) possesses great clinical potential [21]. *ZNF582* is located on chromosome 19q13.43, which contains one KRAB-A-B domain and nine zinc-finger motifs. A recent study of acute myeloid leukemia revealed that aberrant methylation of *ZNF582* is consistent in different disease subtypes, especially in the promoter region. Although the biological function of *ZNF582* is not yet well characterized, a group in Taiwan found similar associations for cervical cancer and adenocarcinoma with *ZNF582* using cervical scrapings. Recently, one study also revealed that hypermethylated *ZNF582* (methylated *ZNF582* (*ZNF582*^*m*^)) performs well as a marker for detecting grade III or higher cervical intraepithelial neoplasia (CIN3+) with 73% sensitivity and 71% specificity for women with low-grade squamous intraepithelial lesions [21-23].

In the present study, we examined the performance of methylated *ZNF582* testing (*ZNF582*^*m*^) for the triage of ASC-US and ASC-H cervical scrapings and compared the results with those of HPV DNA testing.

## Case description

### Patient recruitment

The study was approved by the Department of Clinical Pharmacology, Xiangya Hospital, Central South University, People’s Republic of China. During the period between December 2011 and April 2013, women referred for a colposcopic examination were invited to participate in the study. The eligible women were sexually active, scheduled to undergo a liquid-based cytology test (LBC) within a week, not pregnant, possessed an intact uterus, and no history of treatment for CIN or cervical cancer. The exclusion criteria included women who had a history of cancer related to the reproductive tract, underwent therapy for cervical lesions, received the HPV vaccination, or were currently pregnant. Standard guidelines for the management and treatment of cervical neoplasia were followed in all subjects [24]. The cytology results were classified according to the 2001 Bethesda System [25].

After signing the informed consent, patients immediately received the colposcopic examination and biopsy. The follow-up clinical information was collected. When the biopsy results revealed moderate to severe dysplasia or worse, patients underwent conization or major surgery. The final diagnosis was based on the results of tissue-proven pathology, rather than on the LBC results. To ensure the quality control of the diagnosis, two expert cytologists and two pathologists independently read or reviewed the cytology and histology slides, respectively. All of the patient recruitment and clinical information collection processes were periodically monitored and good clinical practice (GCP) guidelines were followed.

### Specimen collection and DNA preparation

All of the patients had LBC testing before receiving the colposcopic examination. After the informed consent was signed, the LBC containers (Hospitex Diagnostics SRL, Sesto Fiorentino, Italy) were picked up from the Cytology Department, and the residual cervical cells were used for HPV typing and detection of methylated *ZNF582*. All of the molecular tests were performed at the Institute of Clinical Pharmacology, Hunan Key Laboratory of Pharmacogenetics, China, following GLP guidelines. The cells were centrifuged and stored in phosphate-buffered saline (PBS) solution at −20°C from the day of collection, and the assays were finished within 6 months. Genomic DNA (gDNA) was extracted from the collected cells with a QIAamp DNA Mini Kit (Qiagen GmbH, Hilden, Germany) according to the manufacturer’s protocol. A BioSpec-nano spectrophotometer (Shimadzu Corporation, Tokyo, Japan) was used to quantify the amount of extracted DNA.

### DNA methylation tests

For the DNA methylation tests, quantitative methylation-specific PCR (QMSP) using TaqMan-based technologies was performed using the Lightcycler LC480 real-time PCR system (Roche Applied Science, Penzberg, Germany). Briefly, 500 ng of gDNA was subjected to bisulfite conversion using the EZ DNA Methylation-Gold™ Kit (Zymo Research, CA,USA), according to the manufacturer’s recommendations.

**Figure 1:**
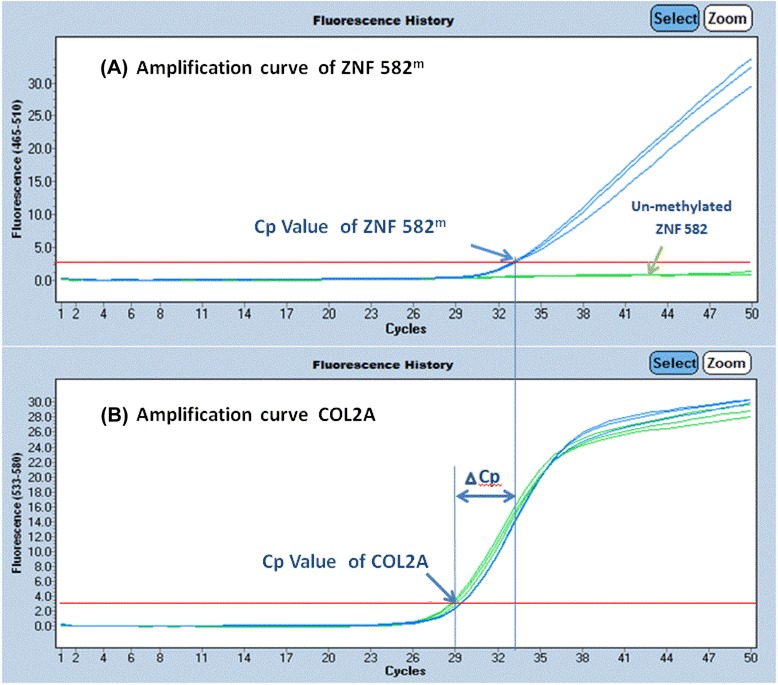
Representative positive and negative *ZNF*582 methylation detection by real-time PCR. For each specimen, *ZNF582* ^*m*^ (labeled with FAM) and *COL2A* (labeled with VIC) were detected simultaneously. Fluorescence data were collected during the annealing/extension step for determination of the crossing point (Cp). The amplification curve for *ZNF582* is shown in panel **(A)** and the control *COL2A* gene is shown in panel **(B)**. The blue arrow indicates the crossing point, and the x-axis value is the so-called Cp value. The ΔCp is the difference between Cp values for *ZNF582* ^*m*^ and *COL2A*. A smaller ΔCp indicates a higher degree of methylation detected in the testing sample. *ZNF582* was deemed to be hypermethylated (or positive) if the ΔCp was smaller than 13 (the cut-off value). The blue arrow is Cp value of *ZNF582* ^*m*^ and the green arrow is the un-methylated *ZNF582* ^*m*^ in panel **(A)**. *ZNF582*, zinc finger protein 582; *ZNF582* ^*m*^, methylated *ZNF582*.

The methylation levels of *ZNF582* were then determined using the qPCR kits developed by iStat Biomedical located in Taiwan. The type II collagen gene (*COL2A*) was designed as the internal reference and tested with each specimen. The crossing point (Cp) value for *COL2A*, which is also the validity indicator of the test, should not be larger than 35. The PCR cycling conditions consisted of an initial incubation at 95°C for 10 min, followed by 50 cycles of denaturation at 95°C for 10 s, and annealing/extension at 60°C for 40 s. Fluorescence data were collected during the annealing/extension step for determination of the Cp. For each sample, two Cp values were obtained: one from the target gene and another from *COL2A*. The DNA methylation level was subsequently determined from the difference between the two Cp values (delta Cp = Cp_*ZNF582*_ - Cp_*COL2A*_). *ZNF582* was deemed to be hypermethylated or positive if the delta Cp was smaller than 13 (the cut-off value). The registered CaSki and A375 cancer cell lines were used as the methylation and no-methylation controls to ensure the quality of bisulfite conversion and qPCR processes. Representative positive and negative QMSP results are shown in Figure 1.

### Laboratory methods for HPV DNA amplification and genotyping

High-risk human papillomavirus (HPV-HR) typing was performed using a nested multiplex PCR (NMPCR) assay that combines degenerate E6/E7 consensus primers and type-specific primers. The identification of HPV-HR was achieved by determining the size of the nested PCR amplification product [26].

### Statistical analysis

The cut-off values for *ZNF582*^*m*^ were generated from the first 100 subjects, including 50 subjects with normal Pap test results and 50 subjects with abnormal Pap test results. Receiver operating characteristic (ROC) curves were generated, and the area under the ROC curve (AUC) was calculated for the detection of the CIN3+ lesions.

SAS software (version 9.2) was used for all statistical analyses (SAS Institute, Ltd., Cary, NC, USA). The chi-square test was used to analyze the status of methylated *ZNF582* or HPV typing in different combinations. Fisher’s exact test was used for the small number data, for example, age under 30 group analysis. For the final analysis, the sensitivity and specificity with a 95% confidence interval (CI) for grade CIN3 lesions or worse were obtained. All differences were considered two-sided and statistically significant at *P* < 0.05.

## Discussion and evaluation

Establishment of routine Pap screening has resulted in a significant decrease in cervical cancer deaths. In China, ASC, including ASC-US and ASC-H, were reported in approximately 10% of papanicolaou smear (Pap smears) [27]. The large number of ASC patients referred for colposcopic examination and follow-up could be an extra burden. In conjunction with the currently available tests, molecular diagnostic tests that can assist in the diagnosis of cancer and that have both a high sensitivity and high specificity would be useful because they would be more accurate than Pap tests alone. Recent guidelines in the USA recommend that adult women with an ASC-US Pap result either have the Pap test repeated at 6 and 12 months, or have an HPV-HR DNA test. In China, repeated follow-up is impractical and time-consuming because many areas do not have sufficient medical resources to perform additional examinations. Another option is to send ASC patients for colposcopy, but colposcopy may not be available in the areas where patients reside thus prohibiting or delaying the diagnosis. Moreover, colposcopy is more invasive and causes anxiety to many women.

Because of the high sensitivity, triage of ASC-US with HPV-HR has been suggested in the cervical screening guidelines. This strategy provides the advantage of missing fewer cases of the severe disease state [28]. However, the low specificity still leads to approximately 50% of women with ASC-US being referred for colposcopy [29]. A similar trend was observed in the present study. The sensitivity and specificity of HPV-HR for detecting CIN3+ were 99.33% and 38.76%, respectively. Seventy-three percent (177/242) of the women with an ASC-US or ASC-H result were positive for HPV-HR, but 61.3% of them had a histological result of CIN2, CIN1, or normal, which is not indicative of a referral for a colposcopic examination. Thus, the HPV-HR test is limited by its low specificity.

In 2012, the American Cancer Society proposed new screening guidelines that included using age-adjusted screening methodologies as well as cytological and HPV co-testing. HPV 16 and 18 typing has been recommended for HPV-HR-positive women [30]. However, geographic variation in the distribution of HPV genotypes exists. In Asia, in addition to HPV types 16 and 18, types 52 and 58 are frequently observed in patients with invasive cervical cancers [31-33]. Similar trends were also observed in the present study even though the test subjects were only limited to women with ASC. In CIN3+ lesions (*N* = 74), HPV 16 (55.4%) and HPV 52 (31.1%) were the most common types, followed by HPV 18 (13.5%), HPV 58 (12.2%), and HPV 33 (9.4%). Among the high percentage of HPV 16 and/or 52 in the CIN3 lesions, the sensitivity, specificity, and accuracy are 74.32%, 73.81%, and 73.97%, respectively. Therefore, the combination of Pap smear and HPV-16/18 typing is not sufficiently accurate for cervical cancer triage in this study.

*ZNF582*^*m*^ has been found to possess clinical potential for the detection of cancer. For example, the frequency of methylated *ZNF582* detection was 100% in SCC tissue. Clinically, *ZNF582*^*m*^ has demonstrated 70% sensitivity and 82% specificity for CIN3+ lesions in a Taiwanese case-control cohort. For triage of LSIL, *ZNF582*^*m*^ also has good sensitivity and specificity with values of 73% and 71%, respectively. Moreover, the *ZNF582*^*m*^ was reported at a high methylation rate in cervical adenocarcinoma, which has been difficult to diagnose.

In triage of the ASC group, *CADM1/MAL*, *WT1*, and other methylation genes have been reported [34]. The present study has proposed a new strategy: co-testing for *ZNF582*^*m*^ and HPV-16/18, in which the patients negative for *ZNF582*^*m*^ undergo HPV-16/18 genotyping. As mentioned, the sensitivity, specificity, and accuracy of the co-test are 89.19%, 70.24%, and 76.03%, respectively. The colposcopy referral rate of the co-test is 47.9%, which is 25.2% less than that of HPV-HR alone. Although eight cases of CIN3+ (10.81%) were missed, nearly 100% of the women with SCC were successfully diagnosed and treated (Table 1). It is also known that the cumulative actuarial rate of progression from CIN3 (severe dysplasia) to CIS or worse is 12% in 4 to 24 months [35,36]. Thus, for patients with ASC undetected by the co-test, a regular follow-up Pap test would be highly recommended. Thus, the progression of the lesion for the eight women with undetected CIN3 would be closely monitored. This triage model would greatly reduce the cost and increase the accuracy for patients residing in countries with limited resources for colposcopy, such as China.

**Table 1:** **Number of colposcopies performed using different triage strategies**

**Triage tests**	**Histological results**	**HPV-HR**	***ZNF582*** ^***m***^ **alone**	***ZNF582*** ^***m***^ **or HPV-16/18**
= < CIN2	168	103 (61.3%)	39 (23.2%)	50 (29.8%)
CIN3+	74	74 (100.0%)	61 (82.4%)	66 (89.2%)
SCC	29	29 (100.0%)	25 (86.2%)	29 (100.0%)
Total colposcopy (referral rate)	242	177 (73.1%)	100 (41.3%)	116 (47.9%)

CIN, cervical intraepithelial neoplasia; HPV-HR, high-risk human papillomavirus; SCC, squamous cell carcinoma; *ZNF582* ^*m*^, methylated *ZNF582*.

The subjects were recruited at the colposcopic examination room. As mentioned, women had results of abnormal pap, inflammation syndrome, cervical erosion, bedding syndromes, or suspected cervical cancer, and these women would be referred to have a colposcopic examination. In our cohort, >90% of women had inflammation syndrome with positive HPV-HR, which correlate with high percentage of CIN2+ (~45%) observed. Although it is known that heterogeneous etiology for cancers and the equivocal nature of ASC, the significance of *ZNF582*^*m*^ in cancer detection was still being observed.

### Characteristics of subjects and colposcopic biopsy results

From November 2011 to March 2013, 242 women were scheduled for colposcopic examinations. The characteristics of the subjects are shown in Table 2. Generally, when women received results of an abnormal Pap, cervical erosion, or suspected cervical cancer, they would be referred to colposcopic examination room from the gynecological outpatient department (OPD). There were 199 women who had ASC-US and 43 who had ASC-H based on the LBC test. The histological and cytological results of the 242 women are shown in Table 2. In total, the colposcopy and biopsy results revealed normal histology in 109 (45.04%) patients, CIN1 in 23 (9.50%) patients, CIN2 in 36 (14.87%) patients, CIN3 in 39 (16.11%) patients, carcinoma *in situ* (CIS) in 6 (2.47%) patients, and invasive carcinoma in 29 (11.98%) patients. Thus, 30.6% (74 of 242) of the total patients had a histological finding of CIN3 or worse (CIN3+). Specifically, 22.1% and 70% of the patients had CIN3+ in the ASC-US and ASC-H groups, respectively.

**Table 2:** **Characteristics and histological results of the study subjects**

	**Histological results**					**Total**
	**Normal**	**CIN 1**	**CIN 2**	**CIN 3/CI**	**SCC/AC**	
Number of subjects						
*N*	109	23	36	45	29	242
(%)	(45.04%)	(9.50%)	(14.88%)	(18.60%)	(11.98%)	(100%)
Age	43.1 ± 10.1 (21.8 to 75.0)					
Mean age	42.39	48.54	39.07	41.77	48.8	43.13
(Min to max)	(22.18 to 74.97)	(28.98 to 66.17)	(21.76 to 64.57)	(27.84 to 57.00)	(33.98 to 65.14)	(21.76 to 74.97)
Cytology results						
ASC-US	103	20	32	29	15	199
(%)	(51.75%)	(10.05%)	(16.08%)	(14.57%)	(7.53%)	(100%)
ASC-H	6	3	4	16	14	43
(%)	(13.95%)	(6.97%)	(9.30%)	(37.20%)	(32.55%)	(100%)
*ZNF582* ^*m*^						
Number	18	8	13	36	25	100
(%)	(16.51%)	(34.78%)	(36.11%)	(80.00%)	(86.20%)	(41.32%)
HPV-HR						
Number	57	13	33	45	29	177
(%)	(52.29%)	(56.52%)	(91.67%)	(100%)	(100%)	(73.14%)

AC, adenocarcinoma of the uterine cervix; ASC-H, atypical squamous cells, high-grade squamous intraepithelial lesions could not be excluded; ASC-US, atypical squamous cells of undetermined significance; CIN, cervical intraepithelial neoplasia; CIS, carcinoma *in situ*; HPV-HR, high risk human papillomavirus; SCC, squamous cell carcinoma.

### Molecular test results in the detection of CIN3+: *ZNF582*^*m*^ and HPV genotyping

The distribution of the molecular tests for different levels of disease severity is listed in Table 2. Table 3 shows the results of the sensitivity, specificity, and accuracy for different molecular tests or combinations of molecular tests in different age groups for the detection of CIN3+ lesions. Among the 242 ASC subjects, 41% were positive for *ZNF582*^*m*^. The sensitivity, specificity, and accuracy of *ZNF582*^*m*^ in the identification of CIN3+ lesion were 82.43%, 76.79%, and 78.51%, respectively. However, when the CIN2 or more severe neoplasms were targeted, the sensitivity and accuracy of *ZNF582*^*m*^ was decreased to 67.27% and 74.38%, respectively, but the specificity was increased to 80.30%. Among women less than 30 years old with ASC, the sensitivity, specificity, and accuracy of *ZNF582*^*m*^ in the identification of a CIN3+ lesion were 75.00%, 81.48%, and 80.65%, respectively. For postmenopausal women with ASC, the sensitivity, specificity, and accuracy were 88.89%, 69.77%, and 75.41%, respectively.

**Table 3:** **The performance of combinations of** ***ZNF582*** ^***m***^ **and HPV tests in detecting CIN3+ in ASC**

**Tests**	**Sensitivity (%)**	**Specificity (%)**	**Accuracy (%)**	**OR (95% CI)**	***P*** **value**
All ages (*N* = 242)					
*ZNF582* ^*m*^	82.43	76.79	78.52	15.52 (7.73 to 31.18)	<0.001
HPV-HR	99.33	38.76	57.37	-	<0.001
HPV-16/18	66.22	88.10	81.41	14.50 (7.42 to 28.37)	<0.001
*ZNF582* ^*m*^ or HPV-16/18	89.19	70.24	76.03	19.47 (8.71 to 43.54)	<0.001
*ZNF582* ^*m*^ or HPV-16/18/52/58	95.95	48.81	63.22	14.79 (6.39 to 34.19)	<0.001
*ZNF582* ^*m*^ or HPV-HR	99.33	34.02	54.10	76.84 (4.68 to 1262.62)	0.002
*ZNF582* ^*m*^ and HPV-HR	82.43	81.55	81.82	-	<0.001
*ZNF582* ^*m*^ and HPV-16/18	59.46	94.64	83.88	25.91 (11.45 to 58.61)	<0.001
Age under 30 (*N* = 31)	Sensitivity (%)	Specificity (%)	Accuracy (%)	OR (95% CI)	*P* value
*ZNF582* ^*m*^	75.00	81.48	80.65	13.20 (1.12 to 154.92)	0.043
HPV-HR	90.00	41.07	48.49	-	0.233
HPV-16/18	75.00	92.59	90.33	37.50 (2.56 to 548.36)	0.009
*ZNF582* ^*m*^ or HPV-16/18	75.00	77.78	77.42	10.50 (0.92 to 120.26)	0.063
*ZNF582* ^*m*^ or HPV-HR	90.00	37.50	45.46	-	0.274
*ZNF582* ^*m*^ and HPV-HR	75.00	85.19	83.87	17.25 (1.42 to 210.12)	0.028
*ZNF582* ^*m*^ and HPV-16/18	75.00	96.30	93.55	78.00 (3.81 to 1595.87)	0.004
Postmenopausal Women	Sensitivity (%)	Specificity (%)	Accuracy (%)	OR (95% CI)	*P* value
*ZNF582* ^*m*^	88.89	69.77	75.41	18.46 (3.70 to 92.14)	<0.001
HPV-HR	97.37	48.86	63.49	-	0.001
HPV-16/18	88.89	86.05	86.89	49.33 (8.97 to 271.23)	<0.001
*ZNF582* ^*m*^ or HPV-16/18	97.37	62.50	73.02	-	<0.001
*ZNF582* ^*m*^ or HPV-HR	97.37	44.32	60.32	-	0.001
*ZNF582* ^*m*^ and HPV-HR	88.89	74.42	78.69	23.27 (4.60 to 117.81)	<0.001
*ZNF582* ^*m*^ and HPV-16/18	77.78	93.02	88.52	46.67 (9.27 to 234.86)	<0.001

*P* values determined by chi-square test or Fisher’s exact test; CI, confidence interval; HPV, human papillomavirus; HPV-HR, high-risk human papillomavirus; OR, odds ratio for CIN3 +; *ZNF582* ^*m*^, methylated *ZNF582*.

The HPV typing indicated that 177 (73.14%) patients were positive for HPV-HR. The most common types of HPV were HPV 16 (23.14%), HPV 52 (23.14%), HPV 58 (14.88%), HPV 68 (7.85%), HPV 33 (7.44%), and HPV 18 (6.05%). The sensitivity and specificity of HPV-HR in identifying CIN3+ were 99.33% and 38.76%, respectively. The percentage of patients who were positive for HPV-16/18 and who had CIN3+ was 66.22% (49 of 74). The prevalence of HPV-16/18 in patients whose tissue was pathologically normal, CIN1, CIN2, CIN3/CIS, or squamous cell carcinoma (SCC) was 7.34%, 17.39%, 22.22%, 55.56%, and 82.76%, respectively.

The results for *ZNF582*^*m*^ combined with different HPV genotyping assays are interesting. The sensitivity of *ZNF582*^*m*^ combined with HPV-HR, HPV-16/18/52/58, or HPV-16/18 was 99.33%, 95.98%, or 89.19%, respectively, but the specificity was increased with values of 34.02%, 48.81%, and 70.24%, respectively.

### Colposcopy referral rate: the performance of *ZNF582*^*m*^ and HPV genotyping in the triage of ASC

**Figure 2:**
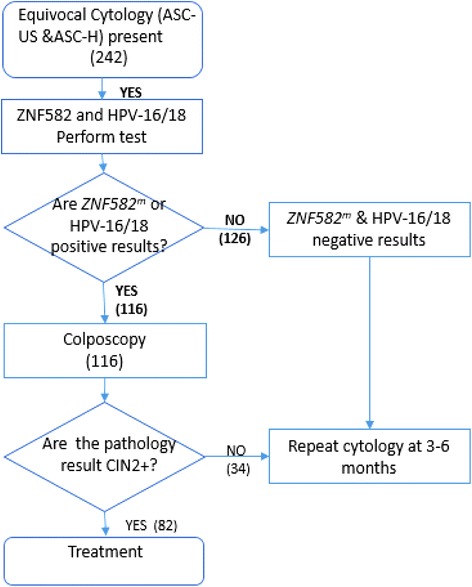
The recommended management of abnormal cervical cytology (Pap smear): a flowchart with methylated *ZNF582* ^*m*^. ASC-H, atypical squamous cells cannot exclude high-grade squamous intraepithelial lesion; ASC-US, atypical squamous cells of undetermined significance; CIN, cervical intraepithelial neoplasia 2; HPV, human papillomavirus-16/18; *ZNF582*, zinc finger protein 582; *ZNF582* ^*m*^, methylated *ZNF582*.

Colposcopy is a diagnostic procedure performed by a physician to examine the cervix, vagina, and vulva carefully for signs of disease. Due to the high cost and insufficient availability of colposcopy, it is desirable to reduce the number of colposcopy referrals while still examining women with severe dysplasia or carcinoma such as CIN3, CIS, or SCC. Our results indicated that the referral rate for colposcopy for *ZNF582*^*m*^ alone, HPV-HR alone, and *ZNF582*^*m*^, or HPV-16/18 are 41%, 73.1%, and 47.9% respectively (Table 1). Previous research has recommended triage procedures using HPV-HR followed by gene methylation screening [34]. Using HPV-HR testing as triage identified no CIN3 and SCC results in the study but the referral rate for colposcopy is 73.1%. If triaged using *ZNF582*^*m*^ testing alone, the colposcopy referral would therefore reduce 31.8% compared to that of HPV-HR. However, only 61 out of the 74 women with CIN3+ and 4 SCC results would be detected. As a result, no treatment would be given to 17 women, who would be in a severe disease stage, especially the four with SCC. To avoid not detecting the severe cases, we have proposed a new strategy to manage ASC patients (Figure 2). This clinical flowchart has incorporated the *ZNF582*^*m*^ and HPV testing into the procedures. Co-testing for *ZNF582*^*m*^ and HPV-16/18 would therefore reduce 61 cases (25.21%) from colposcopy referral (when compared with HPV-HR), yet none of the SCC cases would be missed (Figure 2).

## Conclusions

To the best of our knowledge, the present study is the first to discuss the involvement of *ZNF582*^*m*^ in the ASC group and to consider using the combination of HPV results and methylation markers in the triage of colposcopic referral. It is also the first to compare HPV genotyping and *ZNF582*^*m*^ in the triage of women with ASC Pap smear results. Our results showed that *ZNF582*^*m*^ could be a promising triage technique for women with ASC. We have recommended a new management strategy of abnormal cervical cytology (Pap smear) illustrated in a flowchart. The present study did have some limitations. The subjects were recruited at the colposcopic examination room. Other limitations include using a smaller sample sizes and a lack of extensive long-term follow-up information. However, our results suggest that testing for *ZNF582*^*m*^ followed by HP-V16/18 testing could help to reduce the number of patients with ASC being referred for colposcopy. This could reduce the logistics of obtaining follow-up care, especially in regions where colposcopy is not easily available, which could have a significant impact on public health.

## Abbreviations

ASC-H: Atypical squamous cells cannot exclude high-grade squamous intraepithelial lesions
ASC-US: Atypical squamous cells of undetermined significance
AUC: Area under the curve
CI: Confidence interval
CIN: Cervical intraepithelial neoplasia
CIS: Carcinoma *in situ*
HPV-HR: High-risk human papillomavirus
LBC: Liquid-based cytological test
Pap smear: Papanicolaou smear
PBS: Phosphate-buffered saline
SCC: Squamous cell carcinoma
*ZNF582*: Zinc finger protein 582
*ZNF582*^*m*^: Methylated *ZNF582*
